# Influenza forecasting method based on dual-chan nel feature fusion of VMD decomposition

**DOI:** 10.1038/s41598-026-48594-0

**Published:** 2026-04-24

**Authors:** Hongxin Xue, Chunzi Mengluo, Haijian Liang, Qiqi Jin, Xiaowen Yang, Raoxing Liu

**Affiliations:** 1 College of Computer Science and Technology, North University of China, Taiyuan, 030051 China; 2 Shanxi Provincial Key Laboratory of Machine Vision and Virtual Reality, Taiyuan, 030051 China; 3 Shanxi Province’s Vision Information Processing and Intelligent Robot Engineering Research Center, North University of China, Taiyuan, 030051 China; 4 School of Software, North University of China, Taiyuan, 030051 China; 5 Department of Pulmonary and Critical Care Medicine, First Hospital of Shanxi Medical University, Taiyuan, Shanxi 030001 China

**Keywords:** Influenza Forecasting, Variational Mode Decomposition, Dual-Channel Feature Fusion, Spatio-Temporal Graph Convolutional Network(STGCN), BiGRU-BiLSTM Model, Engineering, Mathematics and computing

## Abstract

Accurate influenza forecasting is critical for timely public health responses and resource allocation. To address challenges such as strong non-stationarity, spatiotemporal heterogeneity, and the prediction lag of traditional models during outbreak peaks , this study proposes a deep learning forecasting framework based on Variational Mode Decomposition (VMD) and Dual-Channel Feature Fusion (VMD-DCFF-IF). The framework first employs VMD to decompose influenza time series into Intrinsic Mode Functions (IMFs) with distinct frequency characteristics, thereby reducing nonlinear coupling. Subsequently, a parallel dual-channel feature extraction network is constructed, utilizing an improved Convolutional Neural Network (CNN) and a Spatio-Temporal Graph Convolutional Network (STGCN) to synergistically capture high-dimensional temporal patterns and spatial correlations. Finally, an adaptive fusion module comprising BiGRU and BiLSTM is designed to achieve dynamic integration of multi-source features for accurate prediction. Historical surveillance data from 2013 to 2023 from the Chinese National Influenza Center were used for validation, comparing the proposed method with mainstream models and advanced Transformer-based architectures such as Informer and Autoformer. Experimental results demonstrate that VMD-DCFF-IF significantly outperforms existing baselines in core metrics, achieving a MASE of 0.508. Phase-specific performance analysis confirms the model’s superior dynamic capturing capability during peak periods, effectively overcoming the overfitting issues common in pure attention mechanisms when processing small-sample, high-noise epidemiological data. Ablation studies further substantiate the unique contributions of each core module to enhancing predictive accuracy and system robustness. To facilitate reproducibility and foster further research, the source code and implementation details are publicly available at https://github.com/xue18334792279/VMD-DCFF-IF/tree/main.

## Introduction

Influenza, an acute respiratory infectious disease caused by influenza viruses, is characterized by a short incubation period, high infectivity, and rapid transmission, posing a severe threat to global public health (WHO 2024)^1^. It is estimated that approximately 1 billion people are infected globally each year, resulting in up to 500,000 deaths. In China, high population density and frequent mobility further increase the risk of large-scale transmission^2,3,7^. Accurate and timely influenza forecasting is a cornerstone of public health prevention and control systems. It provides a valuable time window for formulating control policies, allocating medical resources, and raising public health awareness, thereby mitigating the social and economic impacts of outbreaks^4,5,8^. However, influenza time series data–driven by climate, regional characteristics, and population mobility–exhibit nonlinearity, non-stationarity, sparse sampling, and spatiotemporal coupling. Consequently, the performance of existing models falls far short of practical public health requirements^6–8^. In this context, developing high-precision influenza prediction models by addressing the key limitations of current methods holds significant practical value.

Over the past few decades, influenza forecasting models have evolved from traditional epidemiological models to classical machine learning and, more recently, deep learning approaches. Despite increasing model complexity, existing methods still face critical challenges in addressing the inherent characteristics of influenza data. Traditional epidemiological models (e.g., SIR, SIRS) rely heavily on rigid linear assumptions and fixed differential equation parameters, failing to decouple multi-frequency components such as seasonal trends and random noise; this leads to significant prediction errors during outbreak peaks^9,10^. Classical machine learning models (e.g., RF, XGBoost), while possessing certain non-linear fitting capabilities, are limited by shallow feature extraction and inadequate data preprocessing. By directly processing raw one-dimensional data and neglecting time-frequency decomposition and spatial correlations, their predictive performance remains constrained^11,12^. Even state-of-the-art deep learning models, despite their strong non-linear modeling capabilities, are restricted by single-channel feature extraction. For instance, SAIFlu-Net focuses solely on temporal self-attention^13^, while GAST focuses only on spatial graph attention, with neither achieving a deep fusion of dual spatiotemporal features^14^. Furthermore, these models often employ simple concatenation for feature fusion and lack decomposition processing for non-stationary data, increasing the difficulty of feature extraction. Consequently, even the best-performing GAST model exhibits relatively high prediction errors in practical applications, indicating that existing methods still require improvement.

While existing models have made progress in non-linear fitting, their core limitations remain significant. First, the lack of adaptive decomposition methods for non-stationary influenza sequences makes it difficult to extract effective signals from highly coupled data. Second, there is a dimensional deficiency in spatiotemporal feature mining; previous approaches mostly rely on shallow representations of 1D sequences, overlooking latent spatial interactions and structural features. Finally, feature fusion mechanisms remain relatively rigid, as simple linear concatenation fails to achieve deep complementarity among multi-modal features. These methodological defects underscore the urgent need to develop an end-to-end forecasting framework that integrates data preprocessing, dimensional enhancement, and adaptive fusion to address the complex dynamics of influenza transmission.

To address the aforementioned challenges, this study proposes a Variational Mode Decomposition (VMD)-based Dual-Channel Feature Fusion Influenza Forecasting model (VMD-DCFF-IF). This framework utilizes a multi-stage optimization strategy to systematically resolve critical issues related to data representation, feature extraction, and multi-source fusion. The main contributions of this paper are summarized as follows:

1. Enhanced Data Representation via VMD: To tackle the challenge of insufficient influenza data characterization, we employ Variational Mode Decomposition (VMD) to process raw sequences. By utilizing multi-scale adaptive decomposition techniques, the highly non-stationary original influenza series is decoupled into several Intrinsic Mode Functions (IMFs) with distinct frequency characteristics. This process effectively isolates complex frequency components (e.g., seasonal trends and random noise) that are difficult to identify intuitively in the time domain, thereby reducing signal structural complexity, significantly improving data quality, and laying a solid foundation for subsequent feature extraction.
2. Deep Spatiotemporal Mining based on Dual-Channel Architecture: To address the insufficient mining of latent endogenous features in influenza data, we propose a dual-channel feature extraction mechanism based on time series reconstruction. Graph encoding techniques are utilized to transform one-dimensional IMF components into high-dimensional representations, explicitly embedding both intra-series temporal dependencies and inter-series spatial correlations. Subsequently, a parallel dual-branch network–comprising a 2D Attention-CNN and a Spatio-Temporal Graph Convolutional Network (STGCN)–is employed to achieve comprehensive and deep feature mining of influenza data from both temporal and spatial dimensions.
3. Adaptive Multi-Modal Fusion based on Bi-GLSTM: To overcome the limitations of single-dimensional feature extraction and rigid fusion methods found in existing models, we design an adaptive fusion prediction module (Bi-GLSTM). This module leverages Bidirectional Gated Recurrent Units (BiGRU) to dynamically evaluate feature importance and allocate weights, facilitating the deep integration of spatiotemporal features. Finally, a Bidirectional Long Short-Term Memory (BiLSTM) network utilizes this fused multi-modal information to generate high-precision influenza forecasts.

## Related work

Accurate influenza forecasting has long been a focal point of research in both public health and machine learning. Over the past few decades, researchers have proposed various forecasting models targeting the temporal and spatial characteristics of influenza data. These models can be broadly categorized into three classes: traditional epidemiological models, classical machine learning models, and deep learning models (including state-of-the-art [SOTA] approaches). This section systematically reviews the research progress of these three categories, analyzes the specific limitations of existing methods in conjunction with recent literature, and elucidates the differential innovations of the proposed method within the context of current research.

### Traditional epidemiological models

Traditional epidemiological models constitute the earliest theoretical framework for influenza forecasting, with the Susceptible-Infectious-Recovered (SIR) model and its extensions (SIRS, SEIR) being the most representative^15,16^. Osthus et al.^17^ proposed a state-space SIR model that utilizes a Dirichlet-Beta probability distribution to characterize hyperparameter uncertainty, achieving influenza trend forecasting based on Bayesian inference. Chen et al.^10^ constructed a SIRS model driven by absolute humidity, integrating Kalman filtering techniques to optimize model parameters for forecasting under varying climatic conditions. These models construct dynamic transmission mechanisms by establishing transition relationships between different population groups, offering the advantages of clear physical interpretation and interpretable parameters.

However, in practical applications, such models are constrained by the inherent contradiction between “rigid assumptions and dynamic transmission.” First, modeling based on deterministic differential equations fails to decouple the multi-frequency components within influenza time series (e.g., seasonal trends and random noise), leading to significant prediction errors during peak outbreak periods. Second, these models rely heavily on fixed prior parameters, such as transmission rates, making it difficult to adapt to dynamic shifts in transmission intensity caused by population mobility. To address these rigid defects, this study abandons the fixed-parameter assumption and adopts a Variational Mode Decomposition (VMD) strategy. This method adaptively decomposes non-stationary influenza data into stationary Intrinsic Mode Functions (IMFs) with distinct frequency characteristics, thereby improving the quality of data representation from the preprocessing level.

### Classical machine learning models

With the advancement of statistical learning, classical machine learning models have been widely applied to influenza forecasting due to their robust non-linear fitting capabilities. Representative models include Random Forest (RF), Support Vector Regression (SVR), and Extreme Gradient Boosting (XGBoost)^18–20^. Cheng et al.^11^ employed a Stacking ensemble strategy to combine four machine learning models (RF, SVR, XGBoost), enhancing prediction robustness by selecting high-correlation temporal features. Gantenberg et al. conducted a simulation study to predict seasonal influenza hospitalizations using an ensemble super learner, demonstrating that this ensemble model can effectively improve prediction performance compared with naive predictions.

Despite outperforming traditional methods in non-linear fitting, these models still suffer from unresolved shortcomings in feature engineering. Most models directly input raw one-dimensional case data, lacking effective time-series decomposition, which prevents the sufficient mining of latent internal correlations. Furthermore, feature extraction is often confined to a single temporal dimension, neglecting potential spatial topological relationships between monitoring indicators, resulting in a “flattened” feature representation. To address the issues of single-dimensional extraction and flattened representation, this study designs a “dual-dimensional enhancement strategy” based on IMF components. On one hand, Gramian Angular Difference Fields (GADF) are used to map 1D sequences into 2D images, explicitly embedding temporal dependencies; on the other hand, a spatial graph structure is constructed based on Spearman correlation. This strategy significantly expands the feature space of influenza data, resolving the issue of singular feature representation at its source.

### Deep learning models

In recent years, deep learning models have become the mainstream in influenza forecasting research due to their powerful ability to capture complex non-linear relationships in time series^21–23^. Nikparvar et al.^24^ proposed the MTS-LSTM model, a multivariate ensemble model comprising multiple LSTM components, which utilizes mobile phone data as external covariates to improve forecasting accuracy. Jung et al. proposed the SAIFlu-Net model based on self-attention mechanisms, utilizing LSTM networks to extract regional temporal patterns and modeling dynamic inter-regional dependencies via self-attention. Zhu et al.^14^ designed the Spatio-Temporal Graph Attention Network (GAST), which dynamically captures the heterogeneous effects of different regions over time, enhancing spatial feature mining capabilities.

However, even current SOTA models remain constrained by the dual limitations of “single-channel extraction” and “rigid fusion.” First, existing models often focus on one aspect at the expense of the other; for instance, SAIFlu-Net prioritizes the temporal dimension, while GAST focuses on the spatial dimension, with neither achieving parallel deep extraction of spatiotemporal dual channels. Second, feature fusion typically employs simple concatenation, lacking adaptive weight allocation mechanisms, which causes key features to be submerged in redundant information. Third, even the latest models often overlook the decomposition of non-stationary data, increasing the difficulty of model fitting. To address these limitations, this study constructs a “dual-channel parallel extraction + adaptive fusion” architecture. We utilize an improved CNN-SEnet and a lightweight STGCN to extract spatiotemporal features in parallel, and introduce a BiGRU-BiLSTM fusion module to achieve deep integration of feature-level information through dynamic weight allocation, rather than simple result concatenation.

By systematically reviewing the aforementioned literature, three critical research gaps in the current influenza forecasting field can be distilled, corresponding to the core issues raised in the introduction: (1) Data Level: There is a lack of effective time-frequency decomposition methods for non-stationary, highly coupled data, which is a fundamental cause of instability in prediction accuracy. (2) Feature Level: The mining of latent spatiotemporal features is insufficient, with most models limited to single-dimensional extraction and flattened representations. (3) Fusion Level: There is a lack of adaptive feature fusion mechanisms, as simple concatenation fails to leverage the complementary advantages of multi-modal features.

Currently, no study has constructed an end-to-end framework integrating “time-frequency decomposition, dual-dimensional enhancement, dual-channel extraction, and adaptive fusion.” The proposed VMD-DCFF-IF model is specifically designed to bridge this gap. Our innovation is not merely a simple improvement of individual modules, but a systematic reconstruction of the entire forecasting process–from data preprocessing to feature fusion–aimed at fundamentally resolving the critical limitations of existing models.

## Methodology

The core objective of influenza forecasting is to predict observations $[y_{n+1}, \ldots , y_{n+H}]$ within a future time horizon *H* by learning the mapping relationships from historical influenza time series observations $Y_t = \{y_t \in \mathbb {R} | t=1, 2, \ldots , n\}$ over a time window *T*. The fundamental prediction process is defined as follows:

1 $$\begin{aligned} f: \mathbb {R}^\omega \rightarrow \mathbb {R}^H, \quad [\hat{y}_{n+1}, \dots , \hat{y}_{n+H}] = f(y_{n-\omega +1}, \dots , y_n) + [e_{n+1}, \dots , e_{n+H}] \end{aligned}$$

where $\omega$ represents the length of the input time window, and $[e_{n+1}, \ldots , e_{n+H}]$ denotes the prediction errors within the future time horizon. Consequently, the primary goal of this study is to minimize these prediction errors by optimizing both the model architecture and the data processing pipeline.

**Fig. 1:**
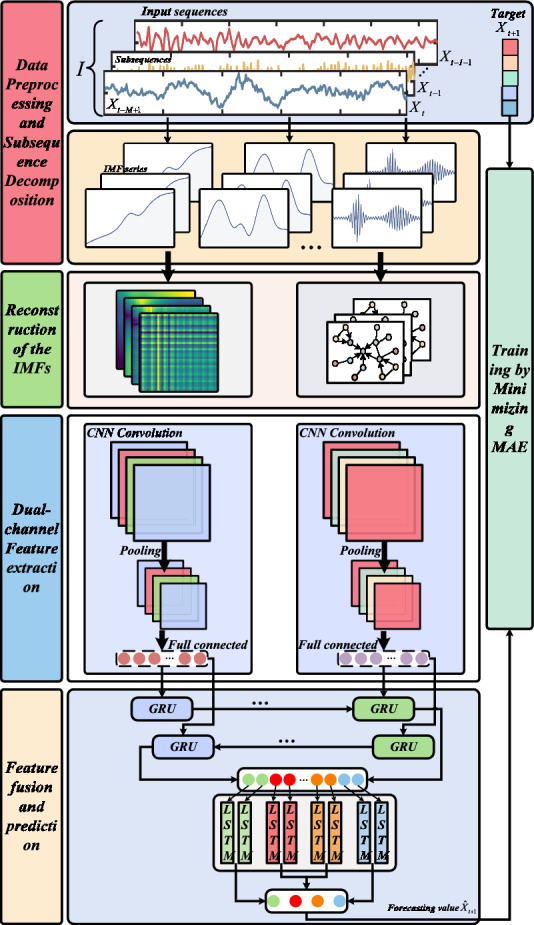
Process framework of two-channel feature fusion method.

To address the challenges of non-stationarity and insufficient spatiotemporal feature mining in influenza forecasting, this study proposes the VMD-DCFF-IF model and establishes an end-to-end data flow framework, as illustrated in Fig. 1. Taking the raw influenza time series as the sole input, the framework forms a cohesive data pipeline comprising four consecutive and interconnected modules: first, Adaptive Decomposition, which decouples the raw sequence via data preprocessing and VMD to reduce spectral complexity; second, Dual-Dimensional Encoding, which implements a dimensionality enhancement strategy to transform one-dimensional components into two-dimensional images and graph structures, thereby explicitly embedding intrinsic correlations; third, Dual-Channel Feature Extraction, which employs parallel deep learning branches to deeply mine spatiotemporal features; and finally, the Bi-GLSTM adaptive fusion module, which integrates multi-modal information via a combined BiGRU and BiLSTM architecture to generate the final prediction results.

## Adaptive sequence decomposition via VMD

To address the non-stationarity and strong coupling characteristics inherent in raw influenza time series, this study employs Variational Mode Decomposition (VMD) as a data preprocessing step to decouple complex frequency components. As a well-established adaptive time-frequency decomposition technique, VMD effectively mitigates the mode mixing limitations associated with Empirical Mode Decomposition (EMD) and its variants. By iteratively optimizing a variational model, it adaptively decomposes the original signal into multiple Intrinsic Mode Functions (IMFs) with distinct frequency features and a residual component^25,26^.

Specifically, for a given raw influenza time series *X*, the VMD algorithm formulates a constrained variational problem by determining the center frequency $\omega _k$ and bandwidth for each IMF component $u_k(t)$, as follows:

2 $$\begin{aligned} \left\{ \begin{aligned} \min _{\{u_k, \omega _k\}}&\left\{ \sum _{k=1}^{K} \left\| \partial _t \left[ \left( \delta (t) + \frac{j}{\pi t} \right) u_k(t) \right] e^{-j\omega _k t} \right\| _2^2 \right\} \\ \text {s.t.} \quad&\sum _{k=1}^{K} u_k = X \end{aligned} \right. \end{aligned}$$

where $\partial _t$ denotes the gradient (bias) operator.

Subsequently, a quadratic penalty term $\alpha$ and a Lagrange multiplier $\lambda$ are introduced to transform the constrained problem into an unconstrained one. The variables $u_k$, $\omega _k$, and $\lambda$ are iteratively updated via the Alternating Direction Method of Multipliers (ADMM) until the convergence condition is satisfied:

3 $$\begin{aligned} \sum _{k} \frac{\left\| u_k^{n+1}(\omega ) - u_k^n(\omega ) \right\| _2^2}{\left\| u_k^{n+1}(\omega ) \right\| _2^2} < \varepsilon \end{aligned}$$

where $\varepsilon$ represents the predefined convergence tolerance. In this study, Permutation Entropy (PeEn) is utilized to determine the optimal number of decomposition modes, set at $K=3$. Consequently, the raw influenza time series is decomposed into three Intrinsic Mode Functions (IMFs) characterized by distinct frequency features–specifically, trend, seasonality, and stochastic noise–along with a residual component. This decomposition effectively reduces the structural complexity of the data, thereby establishing a robust foundation for subsequent feature extraction.

## Dimensionality enhancement of IMF components

To address the issue of flattened feature representation in the one-dimensional IMF components obtained after VMD decomposition, this study implements a dual-dimensional enhancement strategy following the decomposition stage. This strategy aims to transform one-dimensional IMFs into high-dimensional structures to explicitly embed latent spatiotemporal dependencies. Specifically, two complementary dimensionality enhancement strategies are employed to embed the temporal and spatial correlations of the influenza data into the feature space. Both strategies utilize the IMF components as common inputs to facilitate parallel data processing, thereby providing two distinct types of high-dimensional feature data for the subsequent dual-channel extraction module.

### Temporal feature enhancement based on Gramian angular difference field

The Gramian Angular Difference Field (GADF) is a classic time-series imaging technique capable of transforming the temporal correlations of a one-dimensional sequence into pixel information within a two-dimensional image^27^. For a normalized IMF component $\tilde{x}_i \in [0,1]$, GADF first maps the sequence to a polar coordinate system via $r_i = t_i / N$ and $\phi _i = \arccos (\tilde{x}_i)$. Subsequently, it encodes the temporal differences between sequence elements into an image matrix by calculating the sine of the angular difference. The calculation formula is as follows:

4 $$\begin{aligned} \text {GADF} = \sqrt{1-\tilde{x}_i^2} \cdot \tilde{x}_j - \tilde{x}_i \cdot \sqrt{1-\tilde{x}_j^2} \end{aligned}$$

The generated two-dimensional GADF images retain the complete temporal dependencies of the original IMF components and can be directly processed by Convolutional Neural Networks (CNNs) to achieve deep temporal feature extraction.

### Spatial feature enhancement via graph structure construction

To mine the spatial correlations among different IMF components, this study constructs an undirected graph structure based on the Spearman rank correlation coefficient. This process effectively transforms the data representation from a “set of one-dimensional sequences” into a “spatial graph structure.” To enhance the robustness of the graph and filter out redundant noise caused by weak correlations, a correlation thresholding mechanism is introduced. The core steps are as follows:

1. Correlation Calculation: Each IMF component and the raw influenza sequence are treated as graph nodes. The Spearman rank correlation coefficient, denoted as $\rho _s$, is calculated between nodes to quantify their spatial correlations. The formula is given by:
  5 $$\begin{aligned} \rho _s = 1 - \frac{6 \sum _{i=1}^{n} d_i^2}{n(n^2 - 1)} \end{aligned}$$
  where $d_i$ represents the difference in ranks between the two node sequences for the $i-th$ sample(i.e., $d_i = R_{X_i} - R_{Y_i}$, where $R_{X_i}$ and $R_{Y_i}$ are the ranks of the two node sequences at the $i-th$ sample, respectively), and *n* is the total number of samples of the influenza time series.
2. Threshold Filtering and Adjacency Matrix Construction: Constructing a graph directly based on raw correlation coefficients often results in an excessively dense structure, which is prone to causing the over-smoothing problem during graph convolution operations. To address this, a threshold $\delta$ is introduced to filter edge connections. In this study, the threshold is set to $\delta = 0.4$ to retain only correlations of moderate strength or higher. The filtered adjacency matrix *A* is defined as follows:
  6 $$\begin{aligned} A_{ij} = {\left\{ \begin{array}{ll} |\rho _{ij}|, & \text {if } |\rho _{ij}| > 0.4 \\ 0, & \text {otherwise} \end{array}\right. } \end{aligned}$$
  This operation ensures an optimal balance between sparsity and connectivity within the graph structure.
3. Graph Normalization and Feature Embedding: Self-loops are added to the filtered adjacency matrix *A*, followed by degree matrix normalization to obtain the normalized adjacency matrix $\hat{A} = \tilde{D}^{-1/2}\tilde{A}\tilde{D}^{-1/2}$ (where $\tilde{A} = A + I_N$). Finally, the node feature matrix *X* is embedded into the graph space via the graph convolution mapping $M = \hat{A}\text {ReLU}(\hat{A}XW^{(0)})W^{(1)}$, thereby effectively encoding the spatial correlations among the IMF components.

## Dual-channel spatiotemporal feature extraction

Building upon the high-dimensional data generated by the dimensionality enhancement module (GADF images and graph-structured data), this study designs a parallel dual-channel feature extraction architecture characterized by consistent data inputs and synchronized feature outputs. The two channels undergo joint training to achieve independent and comprehensive extraction of the temporal and spatial features of the influenza data. The extracted spatiotemporal features serve as the core output of this stage and are directly passed to the subsequent fusion module.

### Temporal feature extraction channel (improved ForCNN model integrated with SE-Net)

**Fig. 2:**
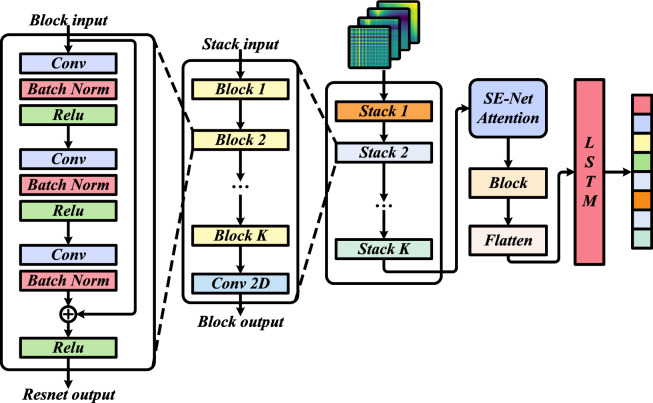
Structure chart of time feature extraction module.

Targeting the two-dimensional images generated via GADF encoding, we construct a temporal feature extraction channel based on an improved ForCNN architecture^28^. This channel aims to capture high-dimensional temporal patterns from the image-based data through the integration of deep convolutional networks and attention mechanisms. As illustrated in Fig. 2, the image sequence is first input into multiple stacked residual blocks, each comprising 2D convolution, Batch Normalization (BN), and a ReLU activation function. Zero-padding is employed to preserve spatial dimensions, while $2\times 2$ downsampling convolution is used to compress parameters and suppress overfitting. To address the degradation problem in deep networks, residual connections are introduced, with the feature mapping relationship defined as:

7 $$\begin{aligned} \bar{x}_{k+1} = h(\bar{x}_k) + F_{\text {block}}(\bar{x}_k) \end{aligned}$$

where $\bar{x}_k$ denotes the input of the *k*-th substructure, $F_{block}$ represents the convolutional transformation, and *h* is the identity mapping.

Subsequently, the extracted feature map *U* is fed into a Squeeze-and-Excitation (SE) module^29^. This module explicitly models channel dependencies to generate an adaptive scaling factor *S*, thereby achieving feature recalibration. This mechanism compels the network to prioritize time intervals rich in critical information. The calculation process is expressed as:

8 $$\begin{aligned} \tilde{X}_{\text {SE}} = F_{\text {scale}}(U, S) = S \cdot U \end{aligned}$$

Finally, the features weighted by the SE module are flattened and fed into an LSTM layer to further extract deep temporal dependencies. The final output of the entire module is formalized as follows, generating a high-dimensional temporal feature matrix $X_t \in \mathbb {R}^{K \times L}$:

9 $$\begin{aligned} \bar{X}_t = \text {LSTM}(\text {flatten}(\text {SE}(F_{\text {final}}))) \end{aligned}$$

### Spatial feature extraction channel (lightweight spatiotemporal graph convolutional network)

**Fig. 3:**
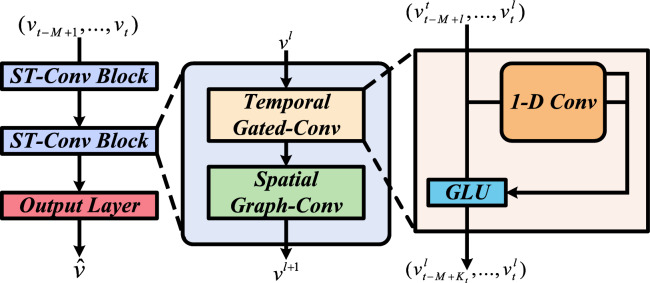
Structure chart of spatial feature extraction module.

Targeting the normalized graph-structured data, this study constructs a spatial feature extraction channel based on a lightweight Spatiotemporal Graph Convolutional Network (STGCN)^30^. As illustrated in Fig. 3, this architecture performs a targeted optimization on the redundant “temporal-spatial-temporal” sandwich structure of traditional STGCNs, focusing primarily on the extraction of spatial correlation features from the graph structure. To maintain temporal causality while capturing spatial dependencies, the channel retains only a single one-dimensional causal convolution block based on Gated Linear Units (GLU). The information flow is controlled through a gating mechanism:

10 $$\begin{aligned} \Gamma *X = P \odot \sigma (Q) \in \mathbb {R}^{C_o \times (M-K_t+1)} \end{aligned}$$

where $\Gamma$ represents the convolution kernel, *P* and *Q* denote the features after channel splitting, $\odot$ indicates the Hadamard product, and $\sigma$ is the Sigmoid activation function. This configuration ensures that the model strictly adheres to temporal causality.

The core spatial feature extraction is implemented through 6 stacked STGCN layers. These layers employ spectral graph convolution approximated via Chebyshev polynomials to aggregate features from nodes and their neighborhoods. The convolution operation is defined as:

11 $$\begin{aligned} S = \sum _{k=0}^{K-1} \theta _k T_k(\tilde{L}) X \end{aligned}$$

where $\tilde{L}$ is the scaled Laplacian matrix, $\theta _k$ is the convolution kernel function, and $T_k$ is the Chebyshev polynomial of order *k*.

To achieve deep mining of spatial features across different receptive fields, the STGCN layers adopt a progressive parameter configuration. The number of filters increases sequentially ($32 \rightarrow 64 \rightarrow 128$), and strides are set alternately to 1 or 2. Furthermore, residual connections are introduced to mitigate information loss in deep networks, ultimately outputting a high-dimensional spatial feature matrix $X_s \in \mathbb {R}^{P \times L}$.

## Bi-GLSTM adaptive fusion and prediction

Addressing the limitation of rigid multi-modal feature fusion mechanisms in traditional models, this study designs the Bi-GLSTM adaptive fusion and prediction module as the terminal stage of the model’s data flow. This module takes the spatiotemporal features extracted by the dual channels as input. By cascading the Bidirectional Gated Recurrent Unit (BiGRU) and the Bidirectional Long Short-Term Memory(BiLSTM) network, it constructs an integrated “fusion-prediction” architecture, realizing a seamless end-to-end connection from feature-level fusion to final influenza prediction^31–34^.

### Multi-source feature concatenation and bigru adaptive fusion

**Fig. 4:**
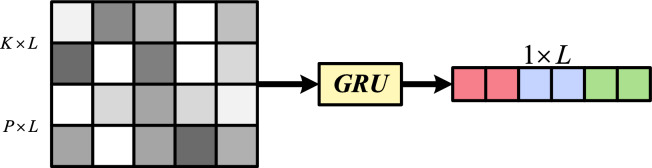
Features splicing.

The fusion process begins with the reorganization of the feature space. First, the temporal feature matrix $X_t \in \mathbb {R}^{K \times L}$ and the spatial feature matrix $X_s \in \mathbb {R}^{P \times L}$, which were extracted in parallel, are concatenated along the channel dimension. This generates a multi-source feature matrix $X_{concat} \in \mathbb {R}^{(K+P) \times L}$ containing complete spatiotemporal information, as illustrated in Fig. 4.

Subsequently, this multi-source matrix is input into the BiGRU network. Unlike simple static linear concatenation, BiGRU utilizes its gating mechanism to dynamically evaluate the importance of spatiotemporal features. Its core update formula is as follows:

12 $$\begin{aligned} h_t = (1 - z_t) h_{t-1} + z_t \tilde{h}_t \end{aligned}$$

where $z_t$ acts as the update gate, determining the retention ratio between historical information $h_{t-1}$ and the current candidate state $\tilde{h}_t$.

Through this mechanism, BiGRU functions as an adaptive weight allocator, capable of dynamically adjusting weights based on the contribution of specific features to the prediction target. Consequently, it outputs a deeply fused high-dimensional feature matrix $X_{fusion}$, effectively resolving the issue where critical features are overshadowed by redundant information.

### BiLSTM temporal modeling and final prediction

The fused features $X_{fusion}$ are directly fed into the BiLSTM network to generate the final prediction. BiLSTM extends the classic LSTM into a bidirectional structure comprising both forward and backward processing layers, enabling the simultaneous capture of past and future contextual information within the sequence. This study employs a two-layer stacked BiLSTM structure (with an output dimension set to 1024) to deeply mine long-term temporal dependencies within the fused features and mitigate the vanishing gradient problem often encountered in long-sequence prediction. Finally, a fully connected layer maps the high-dimensional features into a one-dimensional prediction space, outputting the influenza prediction results $[\hat{y}_{n+1}, \ldots , \hat{y}_{n+H}]$ for the future time window *H*.

## Experimental setup and results analysis

## Dataset description and experimental environment configuration

**Fig. 5:**
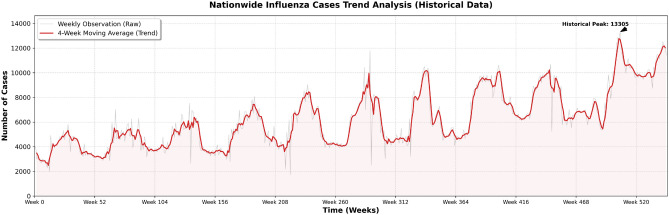
Trend chart of national influenza population.

The dataset for this study is derived from the influenza surveillance database published by the Chinese National Influenza Center (CNIC) (https://ivdc.chinacdc.cn/cnic/). The dataset consists of weekly records of national influenza cases from 2013 to 2023, totaling 546 weekly observation points. As illustrated in Fig. 5, the raw influenza sequence exhibits significant nonlinear fluctuations and seasonal characteristics, accompanied by varying degrees of stochastic noise interference. To verify the generalization capability and predictive performance of the model, the dataset is partitioned into training, validation, and testing sets. Specifically, the final 12 observation points are reserved as the test set for final performance evaluation and are strictly excluded from the training process. The remaining data are processed using a sliding window technique (with a window size set to 36). Among the generated sub-sequences, 80% are utilized for model training, while 20% are used for validation.

In this study, the prediction horizon is set to $H=1$, meaning that historical data from the past 36 weeks are used to forecast influenza incidence for the following week. All experiments were conducted using the TensorFlow 2.9 deep learning framework, with neural network models constructed via the Keras API. The hardware platform is equipped with an Intel i7-12700 CPU (2.10 GHz) and an NVIDIA GeForce RTX 3060 GPU (12 GB VRAM), operating on Windows 11. Detailed information regarding the parameters of the VMD-DCFF-IF model is provided in Table 1. This table encompasses the parameter configurations for the VMD decomposition method as well as the key hyperparameters employed during the model training process. The network architecture of the dual-channel feature extraction model is detailed in Table 2.

**Table 1:** Parameter settings for VMD-DCFF-IF model and training.

Module	Parameter	Value
VMD parameters	$\alpha$	1000
	K	3
	Tol	1e-7
	DC/tau	0
Model training	Optimization Algorithm	Adam
	Learning Rate	1e-5
	Batch Size	128
	Loss Function	MAE

**Table 2:** Network architecture of the VMD-DCFF-IF model.

Module	Network layer	Parameter configuration
Temporal feature extraction module	Stack depth	3
	Number of basic blocks	5
	Convolution kernel	8 filters, kernel size 3 $\times$ 3
	LSTM layer	2 layers, output size 1024
Spatial feature extraction module	STGCN Layer 1	32 filters, kernel size 3 $\times$ 3, stride 1, residual: False
	STGCN Layer 2	32 filters, kernel size 3 $\times$ 3, stride 1, residual: True
	STGCN Layer 3	64 filters, kernel size 3 $\times$ 3, stride 2, residual: True
	STGCN Layer 4	64 filters, kernel size 3 $\times$ 3, stride 1, residual: True
	STGCN Layer 5	128 filters, kernel size 3 $\times$ 3, stride 2, residual: True
	STGCN Layer 6	128 filters, kernel size 3 $\times$ 3, stride 1, residual: True
BiLSTM layer	BiLSTM Layer	2 layers, output size 1024

## Evaluation metrics

This study selects Mean Absolute Scaled Error (MASE) and Symmetric Mean Absolute Percentage Error (sMAPE) as the core evaluation metrics. MASE is scale-insensitive, making it particularly suitable for assessing the prediction quality of non-stationary sequences. sMAPE effectively measures the relative deviation between predicted and actual values and possesses the property of symmetry. Their respective calculation formulas are defined as follows:

13 $$\begin{aligned} MASE = \frac{1}{n} \sum _{t=1}^{n} \left| \frac{y_t - \hat{y_t}}{\frac{1}{n-1} \sum _{i=2}^{n} |y_i - y_{i-1}|} \right| \end{aligned}$$

14 $$\begin{aligned} sMAPE = \frac{1}{n} \sum _{t=1}^{n} \frac{|y_t - \hat{y}_t|}{(|y_t| + |\hat{y}_t|) / 2} \times 100 \end{aligned}$$

where $y_t$ represents the actual observed value at time *t*, $\hat{y}_t$ denotes the predicted value of the model, and *n* is the total number of samples. Lower values for these metrics indicate higher model prediction accuracy.

## Determination of VMD decomposition parameters

### Determination and analysis of the number of VMD modes

In the VMD-DCFF-IF framework, the selection of the number of decomposition modes *K* is of critical importance. An excessively small *K* value leads to mode mixing, failing to isolate signals effectively; conversely, an overly large *K* value introduces spurious modes and noise. To determine the optimal *K*, this study introduces Permutation Entropy (PeEn) as a quantitative metric. PeEn effectively measures the complexity and randomness of a time series, where a lower entropy value indicates higher regularity in the decomposed sub-sequences, which is more conducive to subsequent forecasting. In this study, we calculated the total PeEn of all IMF components within the range of $K \in [2, 15]$, as shown in Table 3. The calculation formula is as follows:

15 $$\begin{aligned} H(m) = -\sum _{j=1}^{K} P_j \ln P_j \end{aligned}$$

**Table 3:** Permutation entropy results under different $K$ values.

$K$’s value	Permutation entropy	$K$’s value	Permutation entropy
2	1.330	9	1.460
**3**	**1.290**	10	1.475
4	1.372	11	1.499
5	1.422	12	1.446
6	1.406	13	1.469
7	1.432	14	1.467
8	1.428	15	1.478

According to the experimental results in Table 3, the PeEn reaches its minimum value of 1.290 when $K=3$, indicating that the sequence complexity and randomness are at their lowest at this point. Therefore, this study sets the number of VMD decomposition modes to $K=3$.

### Sensitivity analysis of the penalty factor $\alpha$

In addition to the number of decomposition modes *K*, the penalty factor $\alpha$ is another critical parameter in the VMD algorithm, as it controls the bandwidth of the resulting Intrinsic Mode Functions (IMFs). Improper selection of $\alpha$ can lead to two main issues: an excessively small value often causes mode mixing, while an overly large value may result in the loss of effective spectral information. To ensure the robustness of the model, this study conducts a sensitivity analysis for the penalty factor $\alpha$, while fixing the optimal number of modes at $K=3$. A set of $\alpha$ values $\{100, 500, 1000, 2000, 5000\}$ is selected, and Permutation Entropy (PeEn) is employed to quantify the regularity of the decomposed components under each configuration.

Table 4 presents the PeEn results for different $\alpha$ values. The data indicates that the PeEn reaches its minimum value of 1.290 when $\alpha =1000$. In the low $\alpha$ range (100-500), the PeEn values remain at a relatively high level (1.467 and 1.354), and the decomposition results suggest that a smaller $\alpha$ imposes loose bandwidth constraints, leading to mode mixing where high-frequency noise infiltrates the low-frequency trend component (IMF1), thereby reducing the stationarity of the extracted features. In the high $\alpha$ range (2000-5000), the PeEn values increase again (1.332 and 1.330), as a larger $\alpha$ exerts excessively strong constraints on the bandwidth and causes an over-smoothing effect, which results in the distortion of the seasonal component (IMF2) and suppresses key amplitude variation features that are vital for accurate influenza prediction. When the optimal $\alpha$ value of 1000 is used, the PeEn of the decomposed signals is the lowest, representing the strongest signal regularity; each IMF achieves clear separation, where IMF1 captures a smooth long-term trend, IMF2 reflects stable seasonal periodicity, and stochastic noise is effectively isolated into the residual component.

**Table 4:** Permutation entropy results under different values of $\alpha$.

$\alpha$ value	Permutation Entropy (PeEn)
100	1.467
500	1.354
**1000**	**1.290**
2000	1.332
5000	1.330

In summary, by combining the quantitative metric of Permutation Entropy (PeEn) minimization with the visual verification of signal orthogonality (see Supplementary Materials for details), this study determines the optimal penalty factor to be $\alpha =1000$.

**Fig. 6:**
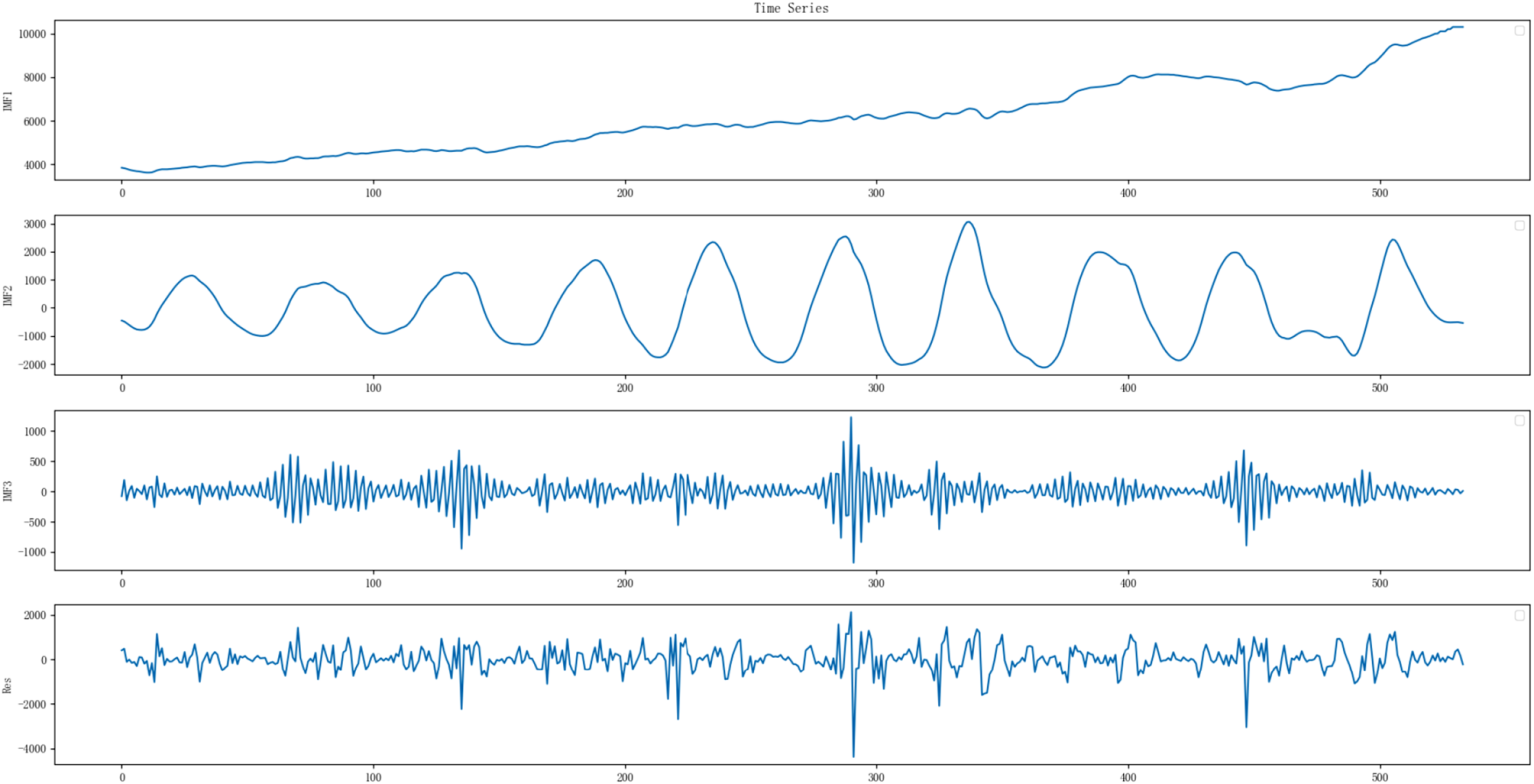
Decomposition diagram of VMD.

The decomposition results based on these parameters are illustrated in Fig. 6. With $K=3$ and $\alpha =1000$, the raw sequence is decomposed into three IMF components and one residual term. Observations indicate that IMF1 exhibits a significant low-frequency trend, reflecting the long-term evolutionary patterns of influenza transmission; IMF2 demonstrates stable periodic fluctuations, which highly coincide with the seasonal outbreak characteristics of influenza; while IMF3 and the residual term contain high-frequency stochastic noise.

## Comparative experimental results and analysis

To verify the superiority of the VMD-DCFF-IF model, this study designed three sets of comparative experiments. The experimental results are summarized in Table 5.

**Table 5:** Forecasting performance comparison of different models on the same dataset.

Experimental group	Model	MASE	sMAPE
1	EMD-DCFF-IF	0.567	3.025
	EEMD-DCFF-IF	0.541	2.866
	CEEMD-DCFF-IF	0.530	2.713
2	SAIIFlu-Net	1.406	12.583
	GAST	0.830	4.669
	TCN+GRU	1.365	13.137
	MTS-LSTM	2.331	13.152
	Informer	0.785	4.120
	Autoformer	0.712	3.890
3	DCFF-IF	0.768	4.067
	VMD-IF	0.556	2.926
	VMD-DCFF	0.513	2.699
	**VMD-D CFF-IF**	**0.508**	**2.688**

Group 1: Validation of Different Decomposition Strategies. This set of experiments fixed the proposed back-end model (DCFF-IF) and only replaced the front-end decomposition method, comparing VMD with EMD, EEMD, and CEEMDAN. The results show that the model employing VMD (the proposed method) achieved the optimal values in both MASE and sMAPE metrics (0.508 and 2.688, respectively), significantly outperforming EMD (0.567/3.025) and its variants. This is primarily attributed to the fact that VMD effectively avoids the mode mixing problem through variational constraints, decomposing non-stationary influenza data into purer and more stable orthogonal components, thereby significantly reducing the forecasting difficulty for the subsequent model.

Group 2: Comprehensive Comparison with SOTA Models. To evaluate the superiority of our model in capturing long-range dependencies and complex spatiotemporal features, this study benchmarks the proposed method against two categories of baseline models: (1) mainstream deep learning architectures specialized for influenza forecasting (GAST, SAIFlu-Net, TCN+GRU, and MTS-LSTM); and (2) state-of-the-art (SOTA) long-sequence forecasting models based on the Transformer framework (Informer and Autoformer). The inclusion of Transformer-based models aims to assess the performance gap between global self-attention mechanisms and our approach within the context of small-sample, high-noise influenza datasets.

As summarized in Table 5, the proposed model achieves the optimal performance across all evaluation metrics. Among the baselines, GAST yields the second-best results (MASE of 0.830) by leveraging Graph Attention Networks for explicit spatial correlation mining. In contrast, traditional architectures like TCN+GRU and MTS-LSTM exhibit significantly higher errors, primarily due to their direct processing of noisy raw data and the absence of targeted multimodal feature fusion. Notably, while Informer and Autoformer demonstrate strong capabilities in long-sequence modeling through self-attention (achieving MASE values of 0.785 and 0.712, respectively), they still slightly underperform compared to our method. This performance gap is likely because Transformer architectures typically require large-scale data to effectively learn complex positional encodings and attention maps, making them susceptible to overfitting on relatively sparse influenza surveillance sequences. Conversely, our method mitigates nonlinear coupling through VMD and explicitly extracts structured features via Graph Convolutional Networks, demonstrating superior robustness and precision under limited data conditions. These results provide compelling evidence that the systematic “Decomposition–Dual-channel Extraction–Adaptive Fusion” strategy is more robust than pure attention mechanisms for non-stationary epidemiological forecasting, surpassing current SOTA models in both the depth and breadth of feature representation.

Group 3: Ablation Study Analysis. To quantify the contribution of each core module, this study conducted a detailed ablation study. The results show that the DCFF-IF model without the VMD decomposition module suffered the most significant performance decline (MASE rose to 2.331), fully demonstrating that data preprocessing is a prerequisite for improving prediction accuracy. The error of the VMD-IF model, which removes the dual-channel structure, also increased (MASE of 0.556), suggesting that focusing solely on the temporal dimension cannot fully capture the complex dynamics of influenza transmission. Additionally, the accuracy of the VMD-DCFF model without the adaptive fusion module decreased slightly (MASE of 0.513), proving that the adaptive weight allocation mechanism of Bi-GLSTM effectively filters key features. In summary, each module in the VMD-DCFF-IF model plays an irreplaceable role, collectively constituting a robust and efficient influenza prediction framework.

**Fig. 7:**
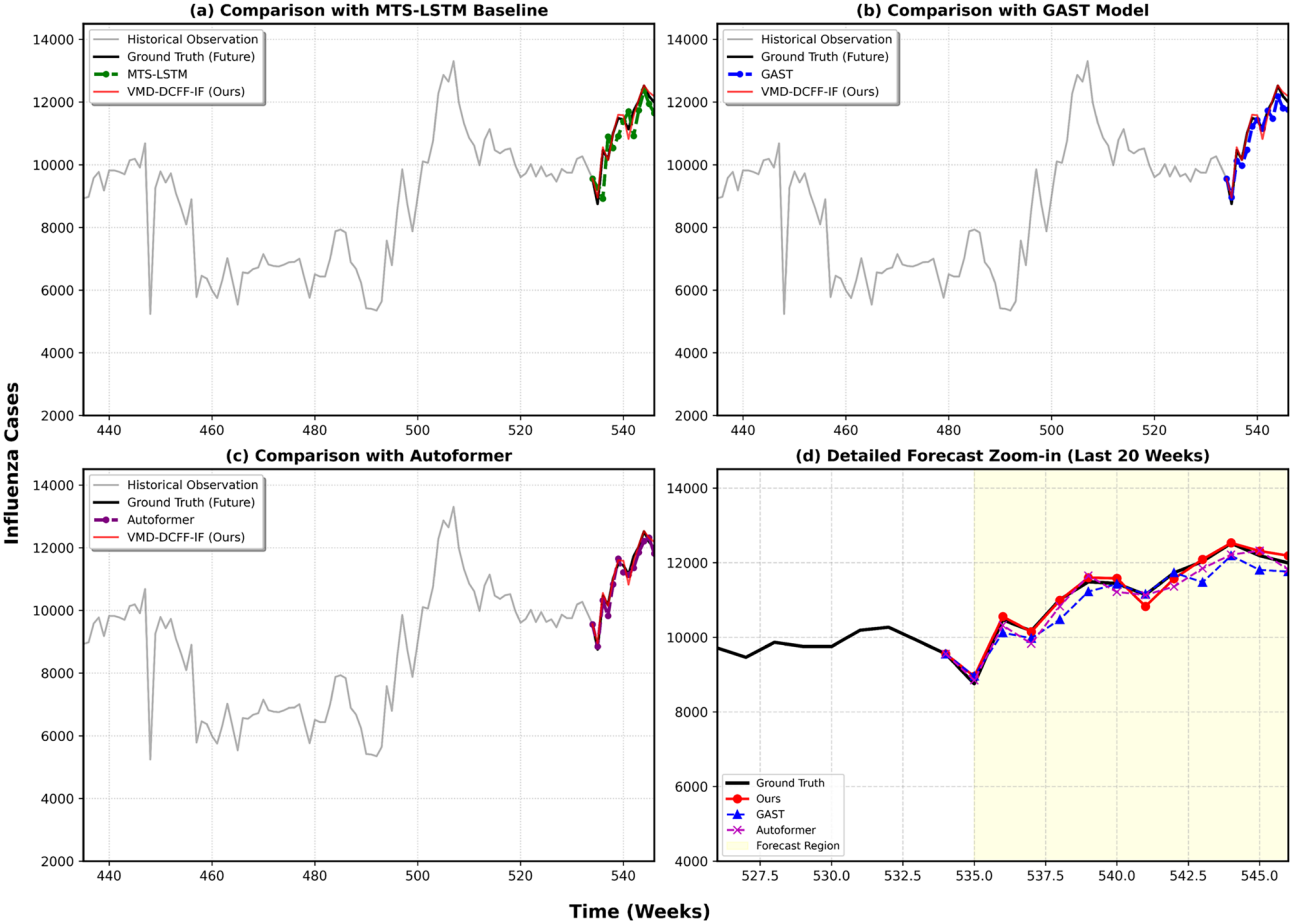
Multi-scenario performance comparison between the proposed model and baseline models on influenza forecasting tasks (**a**) Comparison with MTS-LSTM baseline; (**b**) Comparison with GAST model (Red line indicates ours); (**c**) Comparison with Autoformer model (Red line indicates ours); (**d**) Zoom-in details of the forecast interval (Last 12 weeks).

To further intuitively verify the effectiveness of the aforementioned numerical evaluation metrics and deeply analyze the dynamic capturing capabilities of different models during the actual evolution of influenza, this paper selects a continuous influenza sequence of 100 weeks (weeks 447 to 546), including the test set, for visual comparative analysis, and the results are shown in Fig. 7. In the figure, the solid gray line represents the historical observation data of the first 88 weeks to provide the background trend of influenza evolution; the solid black line represents the ground truth of influenza trends for the final 12 weeks; each colored dashed line corresponds to the output results of different models within the prediction interval of the final 12 weeks. In this way, the capturing accuracy of each model regarding future short-term sudden fluctuations after undergoing long-term historical trend evolution can be clearly observed.

Figure 7 illustrates the comparison of dynamic capturing capabilities among various models within the context of the test set and relevant observations. Through the observation of Fig. 7a, it can be found that although the MTS-LSTM model (green dashed line) can track the overall evolutionary trend of influenza data, its prediction curve exhibits a significant “Lag Effect”. The changes in predicted values clearly lag behind the sudden mutations of the real values, indicating that the model has limitations in utilizing time-series contextual information to predict trend turning point s.

In the comparative analysis against state-of-the-art (SOTA) baseline models (Fig. 7b,c), the VMD-DCFF-IF model (red solid line) of this paper is specifically superimposed as a reference to intuitively present the differences in fitting details. The results show that while the GAST model (blue dashed line) outperforms MTS-LSTM in trend sensitivity, its fitting accuracy at key extreme points such as peaks and troughs is still inferior to our model, with a certain systematic bias (Fig. 7b). At the same time, the Autoformer model (purple dashed line) demonstrates strong instability due to the sparse nature of small-sample influenza data, with unreasonable oscillation phenomena occurring in local areas; in contrast, the VMD-DCFF-IF model over the same period always maintains a high degree of fit with the real curve, exhibiting stronger robustness (Fig. 7c).

The partial enlargement of the data for the final 12 weeks within the prediction interval (Fig. 7d) further confirms that the overlap between our model (red dotted line) and the real values (black solid line) is the highest among all compared models, essentially achieving precise tracking with zero phase difference. This visualization result powerfully proves that the VMD-DCFF-IF model, by virtue of its unique dual-channel feature fusion mechanism, can effectively overcome the common lag and oscillation problems of traditional models, maintaining extremely high fidelity and reliability in short-term sudden fluctuation prediction within a long-term prediction context.

## Phase-specific performance analysis

The transmission of influenza virus exhibits significant seasonality and abruptness, and the predictive performance of a model across different epidemic phases directly determines its practical value in public health early warning. To deeply verify the temporal robustness of the model, this paper divides the test set into two phases–Peak Period and Stable Period–based on influenza epidemiological characteristics. Among them, the Peak Period corresponds to the periods of rapid surge and high-level operation of case numbers (such as winter and spring peaks), exhibiting intense non-stationarity and nonlinearity; the Stable Period refers to the stage where case numbers are at a low level with gentle fluctuations.

The two baseline models with the strongest overall performance (Autoformer and GAST) were selected for comparison with the VMD-DCFF-IF model proposed in this paper, and the results are shown in Table 6.

**Table 6:** Performance comparison of models during different influenza epidemic periods (Peak vs. Stable).

Model	Phase	MASE	sMAPE
Autoformer	Peak	0.825	4.450
	Stable	0.650	3.510
GAST	Peak	0.940	5.120
	Stable	0.780	4.210
VMD-DCFF-IF	Peak	**0.545**	**2.890**
	Stable	**0.480**	**2.510**

Experimental results show that the errors of all models in the stable period are lower than those in the peak period, which conforms to the high volatility characteristics of the influenza peak period. However, the MASE of the VMD-DCFF-IF model in the peak period is only 0.545, demonstrating a significant advantage compared to Autoformer (0.825) and GAST (0.940). This is primarily attributed to the fact that VMD decomposition effectively separates the ultra-high frequency components representing sudden fluctuations from the low-frequency components representing long-term trends, enabling the dual-channel network to capture dynamic turning points with greater focus, thereby maintaining extremely high tracking capability even during epidemic high-incidence periods. Furthermore, by comparing the index differences between the two stages, it can be observed that the performance variance of the proposed model is the smallest. Although Transformer-like models perform adequately when the data distribution is stable, their prediction bias increases significantly when facing the intense Distribution Shift during peak periods.

**Fig. 8:**
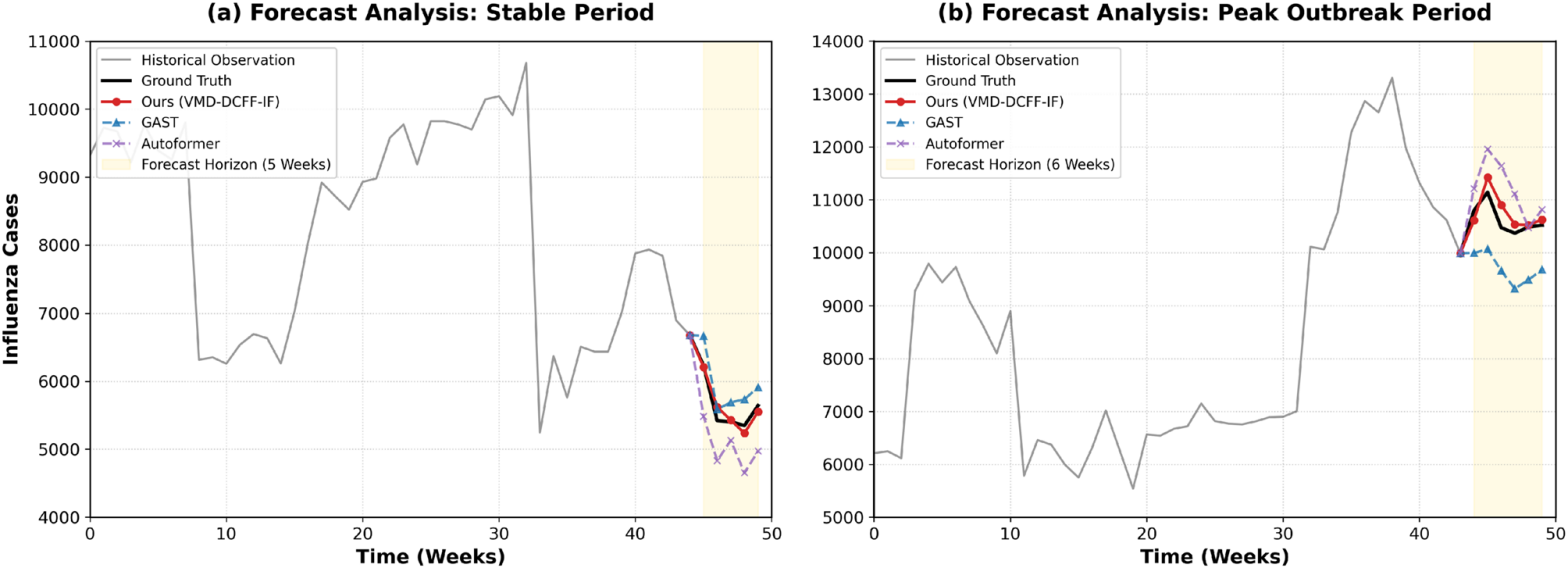
Comparison of prediction performance between the proposed model and baselines during different influenza epidemic periods. (**a**) Forecasting results in the stable period; (**b**) Forecasting results in the peak outbreak period.

To intuitively analyze the dynamic adaptation capability of the models in the face of the non-stationary characteristics of the data, this paper conducts a visual comparative analysis of the stable period and the peak outbreak period (as shown in Fig. 8). In the figure, the solid gray line represents the historical observation sequence, the solid black line depicts the ground truth of the trends within the prediction interval, and the red solid line displays the inference results of the proposed model.

As shown in Fig. 8a, in the Stable Period, although the number of influenza cases is at a relatively low level, fluctuations still exist. In this scenario, the baseline models exhibit obvious biases: the GAST model (blue dashed line) tends to overestimate the actual number of cases, while the Autoformer model (purple dashed line) fails to fully capture the frequency of the fluctuations. In contrast, the VMD-DCFF-IF model (red solid line) of this paper demonstrates excellent calibration capability, accurately fitting the ups and downs of the real curve.

As shown in Fig 8b, in the Peak Outbreak Period, the number of influenza cases climbs rapidly and forms a sharp peak. The performance differences of various models are further amplified at this stage: the GAST model exhibits a clear “conservative” tendency, seriously underestimating the intensity of the peak (Underestimation); while Autoformer, although sensitive in response, experiences an Overshoot phenomenon, with predicted values significantly exceeding the real peak. Benefiting from the effective extraction of high-frequency mutation signals by the VMD dual-channel mechanism, the model in this paper suppresses noise while retaining sensitivity to mutation trends. It not only accurately predicts the peak height but also maintains high synchronization with the real evolution in terms of phase, powerfully proving its Temporal Robustness in complex influenza surveillance tasks.

In summary, the VMD-DCFF-IF model possesses good adaptability and robustness across different epidemic stages, capable of providing more reliable decision support for actual influenza prevention and control.

## Computational complexity and practicality analysis

Table 7 provides a detailed comparison of the performance of VMD-DCFF-IF and various baseline models across key indicators, such as the number of parameters (Parameters), training time per epoch (Training Time per Epoch), and inference latency (Inference Latency).

**Table 7:** Comparison of computational complexity and engineering practicality metrics among models.

Model	Params (M)	Training time (s/epoch)	Inference time (ms/sample)	MASE
MTS-LSTM	4.25	2.1	4.5	2.331
GAST	2.84	5.8	15.2	0.830
Autoformer	12.50	14.2	28.4	0.712
VMD-DCFF-IF (Our)	28.65	18.5	42.1	0.508

From the perspective of the trade-off between model capacity and prediction accuracy, the parameter count of VMD-DCFF-IF is approximately 28.65 M. This increase in computational load primarily stems from the high-dimensional mapping space established within the dual-channel architecture for deep feature extraction, such as the BiGRU and BiLSTM layers with an output dimension of 1024, as well as the deeply integrated CNN and GNN modules. Experimental data indicate that although model architectures like MTS-LSTM and GAST are lighter (with parameter counts of 4.25 M and 2.84 M, respectively), their limited feature representation capabilities restrict their ability to capture complex influenza dynamics, leading to higher prediction errors. Even Autoformer, which possesses 12.50 M parameters, shows accuracy significantly lower than the proposed model due to a lack of structural constraints targeting the non-stationary characteristics of influenza sequences. This comparison confirms the necessity of moderately increasing computational space in exchange for prediction accuracy, enabling the proposed model to ultimately achieve the optimal MASE value of 0.508.

In terms of engineering practicality, the single-sample inference latency of VMD-DCFF-IF in an RTX 3060 GPU environment is only 42.1 ms. Given that influenza surveillance typically uses a “week” as the prediction granularity, sub-second inference responses are fully capable of meeting the timeliness requirements of clinical applications and public health decision-making. Furthermore, although the training time per epoch for this model (18.5 s) is slightly higher than that of the baseline models, considering that model retraining in practical applications usually adopts an offline mode (such as monthly or quarterly updates), this will not create a bottleneck for the deployment of real-term early warning services. In summary, while maintaining high-performance prediction, VMD-DCFF-IF achieves a favorable balance between computational efficiency and engineering deployment.

## Conclusion

To address the challenges of “data non-stationarity” and “insufficient spatiotemporal feature capture” in influenza forecasting, this research proposes the VMD-DCFF-IF framework. Diverging from traditional models that rely on raw sequences or single-dimensional feature extraction, our approach establishes a systematic “Decomposition–Encoding–Extraction–Fusion” paradigm. Based on experimental validation using China National Influenza Surveillance data (2013-2023), the conclusions are as follows:

First, experiments demonstrate that Variational Mode Decomposition (VMD) effectively decouples complex influenza sequences into stationary orthogonal components, significantly outperforming methods like EMD in reducing spectral complexity. By integrating GADF imaging and graph structure construction, the model successfully transforms 1D sequences into high-dimensional representations. Combined with a dual-channel architecture and an adaptive fusion mechanism, it achieves precise selection of multi-modal features. VMD-DCFF-IF surpasses advanced baselines, including GAST, SAIFlu-Net, and self-attention-based models like Informer and Autoformer, across key metrics. Findings indicate that while Transformer-like architectures are prone to overfitting on the small-sample data typical of influenza surveillance, our model achieves superior accuracy by mitigating nonlinear coupling through VMD preprocessing.

Second, phase-specific performance analysis further validates the model’s temporal robustness. During influenza peak periods, VMD-DCFF-IF effectively captures drastic fluctuations in case numbers, achieving precise tracking with virtually no phase lag–thereby alleviating the “lag effect” and peak fitting deficiencies prevalent in traditional deep learning models. During stable periods, the model maintains exceptionally high fitting fidelity. Additionally, computational cost analysis shows that despite its larger dual-channel architecture, the single-sample inference latency on a GPU is only 42.1 ms. Given the weekly update frequency of influenza surveillance, this overhead is well within acceptable limits, meeting the engineering requirements for real-time early warning systems in disease control departments.

Despite the promising results, the current construction of the spatial graph structure relies primarily on statistical correlation, which may not fully capture dynamic causal relationships. Future work will focus on integrating multi-source heterogeneous data (e.g., meteorological monitoring and population mobility) to enhance model interpretability, while exploring dynamic graph learning techniques to further extend the accuracy of early warnings over longer forecasting horizons.

## Supplementary Information

Supplementary Information.

## Data Availability

The influenza surveillance data used in this study are publicly available from the Chinese National Influenza Center (CNIC) at https://ivdc.chinacdc.cn/cnic/.

## References

[1] Wang, B. Surveillance on influenza-like illness in Hongqiao district of Tianjin from 2011 to 2020. *Strait J. Prev. Med.* **27**, 31–34 (2022).

[2] Yamin, M. Counting the cost of covid-19. *Int. J. Inf. Technol.* **12**, 311–317. 10.1007/s41870-020-00466-0 (2020). 32412538 10.1007/s41870-020-00466-0 PMC7220645

[3] Chai, G., Wang, B. & Sha, Y. Public health risk forecasting with multiple machine learning methods combined: Case study of influenza forecasting in Lanzhou, China. *Data Anal. Knowl. Discovery* **5**, 90–98 (2021).

[4] Biggerstaff, M. et al. Results from the second year of a collaborative effort to forecast influenza seasons in the united states. *Epidemics* **24**, 26–33. 10.1016/j.epidem.2018.02.003 (2018). 29506911 10.1016/j.epidem.2018.02.003 PMC6108951

[5] Lages, N. C. et al. Dynamic risk perceptions in times of avian and seasonal influenza epidemics: A repeated cross?sectional design. *Risk Anal.* **41**, 2016–2030. 10.1111/risa.13706 (2021). 33580509 10.1111/risa.13706

[6] Ma, P. et al. The association between influenza onset and meteorological inducers in Shenzhen and construction of predictive models. *Acta Meteorol. Sin.* **80**, 421–432. 10.11676/qxxb2022.039 (2022).

[7] Han, Y.-Y. et al. Spatial and temporal distribution characteristics of seasonal a (h3n2) influenza in China, 2014–2019. *Chin. J. Epidemiol.* **44**, 937–941 (2023). 10.3760/cma.j.cn112338-20221212-01059 37380416

[8] Liu, Y., Huang, Q., Zhang, L., Wang, C. & Liu, Y. Spatial-temporal analysis and prediction model for the incidence of influenza from 2004 to 2018 in China. *Chin. J. Disease Control Prevent.* **27**, 176–183 (2023).

[9] Cooper, I., Mondal, A. & Antonopoulos, C. G. A sir model assumption for the spread of covid-19 in different communities. *Chaos Solitons Fractals* **139**, 110057. 10.1016/j.chaos.2020.110057 (2020). 32834610 10.1016/j.chaos.2020.110057 PMC7321055

[10] Chen, X. et al. Forecasting influenza epidemics in China using transmission dynamic model with absolute humidity. *Infect. Dis. Model.* **10**, 50–59 (2025). 39319283 10.1016/j.idm.2024.08.003 PMC11419822

[11] Cheng, H.-Y. et al. Applying machine learning models with an ensemble approach for accurate real-time influenza forecasting in Taiwan: Development and validation study. *J. Med. Internet Res.* **22**, e15394. 10.2196/15394 (2020). 32755888 10.2196/15394 PMC7439145

[12] Gantenberg, J. R. et al. Predicting seasonal influenza hospitalizations using an ensemble super learner: A simulation study. *Am. J. Epidemiol*. **192**, 1688–1700 (2023). 37147861 10.1093/aje/kwad113 PMC10558190

[13] Jung, S., Moon, J., Park, S. & Hwang, E. Self-attention-based deep learning network for regional influenza forecasting. *IEEE J. Biomed. Health Inform.* **26**, 922–933. 10.1109/jbhi.2021.3093897 (2022). 34197330 10.1109/JBHI.2021.3093897

[14] Zhu, X. et al. Modeling epidemic dynamics using graph attention based spatial temporal networks. *PLoS ONE* **19**, e0307159 (2024). 39008489 10.1371/journal.pone.0307159 PMC11249270

[15] de Souza, D. B. et al. Fock-space approach to stochastic susceptible-infected-recovered models. *Phys. Rev. E* **106**, 014136. 10.1103/physreve.106.014136 (2022). 35974542 10.1103/PhysRevE.106.014136

[16] Sidi Ammi, M. R. et al. Global analysis of a time fractional order spatio-temporal SIR model. *Sci. Rep.* **12**, 5751. 10.1038/s41598-022-08992-6 (2022). 35388030 10.1038/s41598-022-08992-6 PMC8984679

[17] Osthus, D., Hickmann, K. S., Caragea, P. C., Higdon, D. & Del Valle, S. Y. Forecasting seasonal influenza with a state-space sir model. *Ann. Appl. Stat.* **11**, 202. 10.1214/16-aoas1000 (2017). 28979611 10.1214/16-AOAS1000 PMC5623938

[18] Cho, Y. et al. Prediction of hospital-acquired influenza using machine learning algorithms: a comparative study. *BMC Infect. Dis.* **24**, 1–11 (2024). 38698304 10.1186/s12879-024-09358-1 PMC11067145

[19] Xue H. X., et al. Influenza trend prediction method combining Baidu index and support vector regression based on an improved particle swarm optimization algorithm. *AIMS Math.* **8**, 25528–25549 (2023).

[20] Levi, Y., Brandeau, M., Shmueli, E. & Yamin, D. Prediction and detection of side effects severity following covid-19 and influenza vaccinations: utilizing smartwatches and smartphones. *Sci. Rep.* **14**. 10.1038/s41598-024-56561 (2021). 10.1038/s41598-024-56561-w PMC10933398 38472345

[21] Lee, K., Ray, J. & Safta, C. The predictive skill of convolutional neural networks models for disease forecasting. *PLoS ONE* **16**, e0254319. 10.1371/journal.pone.0254319 (2021). 34242349 10.1371/journal.pone.0254319 PMC8270135

[22] Dhaka, P. & Nagpal, B. WOM-based deep BILSTM: smart disease prediction model using WOM-based deep BILSTM classifier. *Multimed. Tools Appl.* **82**, 25061–25082 (2023).

[23] Papagiannopoulou, E., Bossa, M. N., Deligiannis, N. & Sahli, H. Long-term regional influenza-like-illness forecasting using exogenous data. *IEEE J. Biomed. Health Inform.* **28**, 3781–3792 (2024). 38483802 10.1109/JBHI.2024.3377529

[24] Nikparvar, B., Rahman, M. M., Hatami, F. & Thill, J.-C. Spatio-temporal prediction of the covid-19 pandemic in us counties: Modeling with a deep LSTM neural network. *Sci. Rep.* **11**, 21715 (2021). 34741093 10.1038/s41598-021-01119-3 PMC8571358

[25] Dragomiretskiy, K. & Zosso, D. Variational mode decomposition. *IEEE Trans. Signal Process.* **62**, 531–544. 10.1109/tsp.2013.2288675 (2014).

[26] Ding, T. et al. Prediction of significant wave height using a VMD-LSTM-rolling model in the south sea of China. *Front. Mar. Sci.* **11**, 1382248 (2024).

[27] Shen, J., Wu, Z., Cao, Y., Zhang, Q. & Cui, Y. Research on fault diagnosis of rolling bearing based on Gramian angular field and lightweight model. *Sensors* **24**, 5952 (2024). 39338697 10.3390/s24185952 PMC11435585

[28] Semenoglou, A.-A., Spiliotis, E. & Assimakopoulos, V. Image-based time series forecasting: A deep convolutional neural network approach. *Neural Netw.* **157**, 39–53. 10.1016/j.neunet.2022.10.006 (2023). 36306658 10.1016/j.neunet.2022.10.006

[29] Hu, J., Shen, L., Albanie, S., Sun, G. & Wu, E. Squeeze-and-excitation networks. *IEEE Trans. Pattern Anal. Mach. Intell.*10.1109/tpami.2019.2913372 (2020). 31034408 10.1109/TPAMI.2019.2913372

[30] Wang, C., Zhang, K., Wang, H. & Chen, B. Auto-stgcn: Autonomous spatial-temporal graph convolutional network search. *ACM Trans. Knowl. Discov. Data* **17**, 1–21. 10.1145/3571285 (2023).

[31] Li, D., Li, W., Zhao, Y. & Liu, X. The analysis of deep learning recurrent neural network in english grading under the internet of things. *IEEE Access* (2024).

[32] Duan, Y., Liu, Y., Wang, Y., Ren, S. & Wang, Y. Improved bigru model and its application in stock price forecasting. *Electronics* **12**, 2718. 10.3390/electronics12122718 (2023).

[33] Jang, H. et al. Bi-directional long short-term memory neural network modeling of data retention characterization in 3-d triple-level cell nand flash memory. *IEEE Trans. Electron Devices* **69**, 4241–4247. 10.1109/ted.2022.3182282 (2022).

[34] Zhang, X., Yang, Y., Liu, J., Zhang, Y. & Zheng, Y. A CNN-BILSTM monthly rainfall prediction model based on SCSSA optimization. *J. Water Climate Change* **15**, 4862–4876 (2024).

