# Supplementary material for: Influenza forecasting method based on dual-chan nel feature fusion of VMD decomposition
Source: Sci Rep. 2026 Apr 24;16:19073. doi: 10.1038/s41598-026-48594-0 (PMC13280402; doi:10.1038/s41598-026-48594-0)
Supplement: Supplementary file 1 — Supplementary Information. [file 41598_2026_48594_MOESM1_ESM.docx]

**
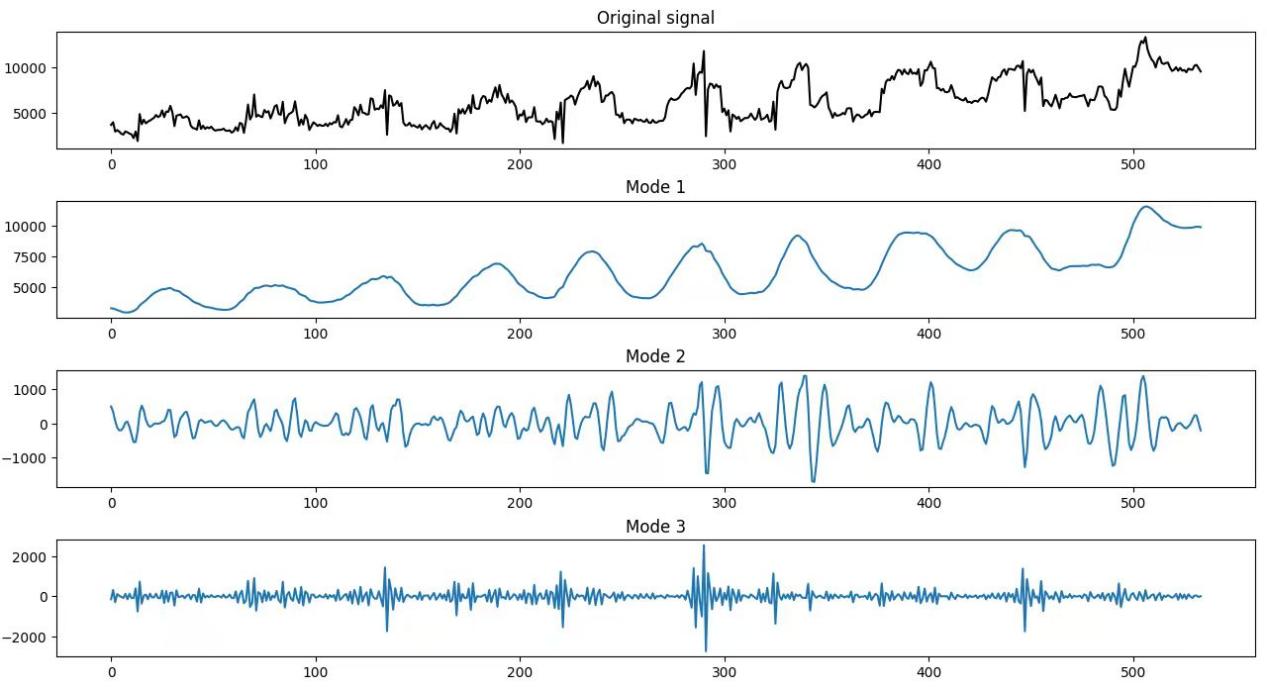
**

**（a）VMD decomposition plot for α= 100**

**
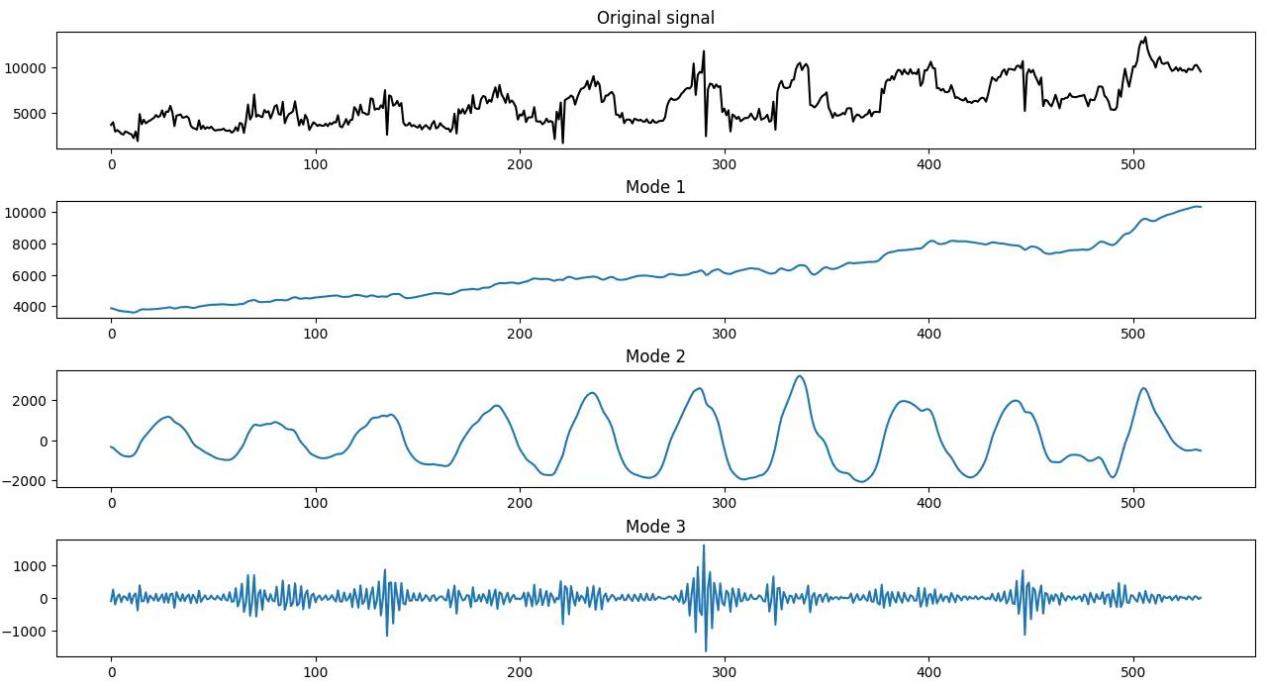
**

**（b）VMD decomposition plot for α= 500**

**
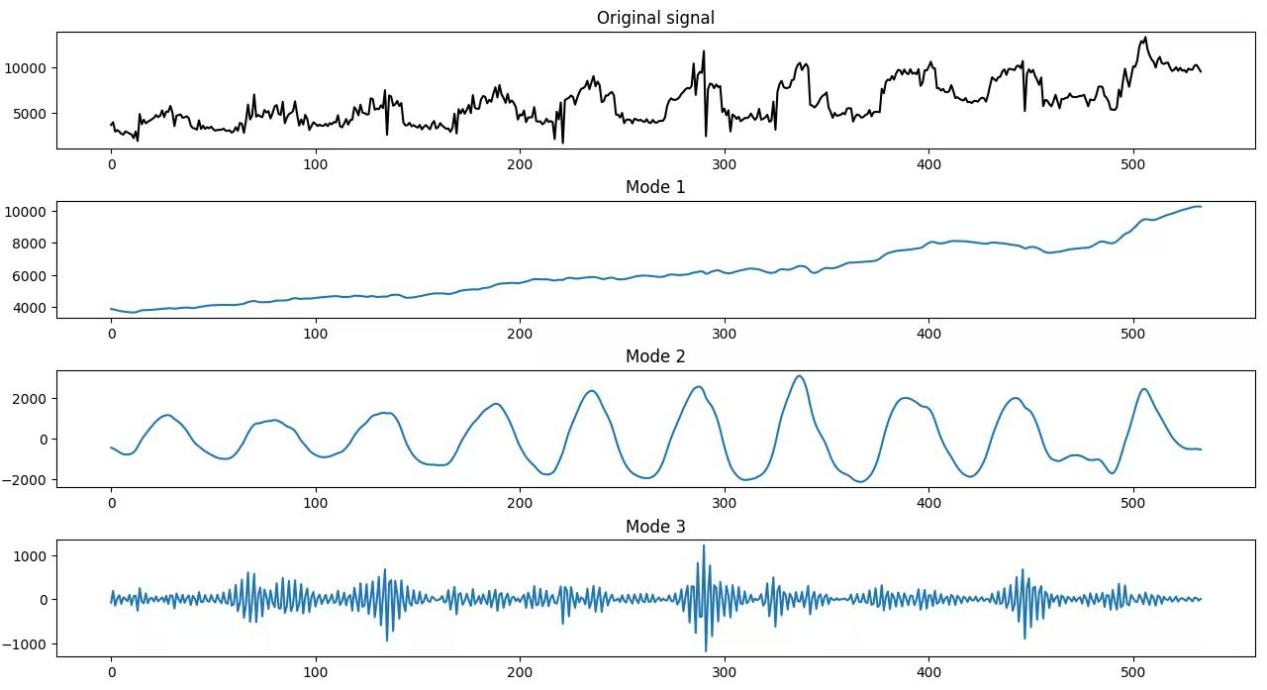
**

**（c）VMD decomposition plot for α= 1000**

****
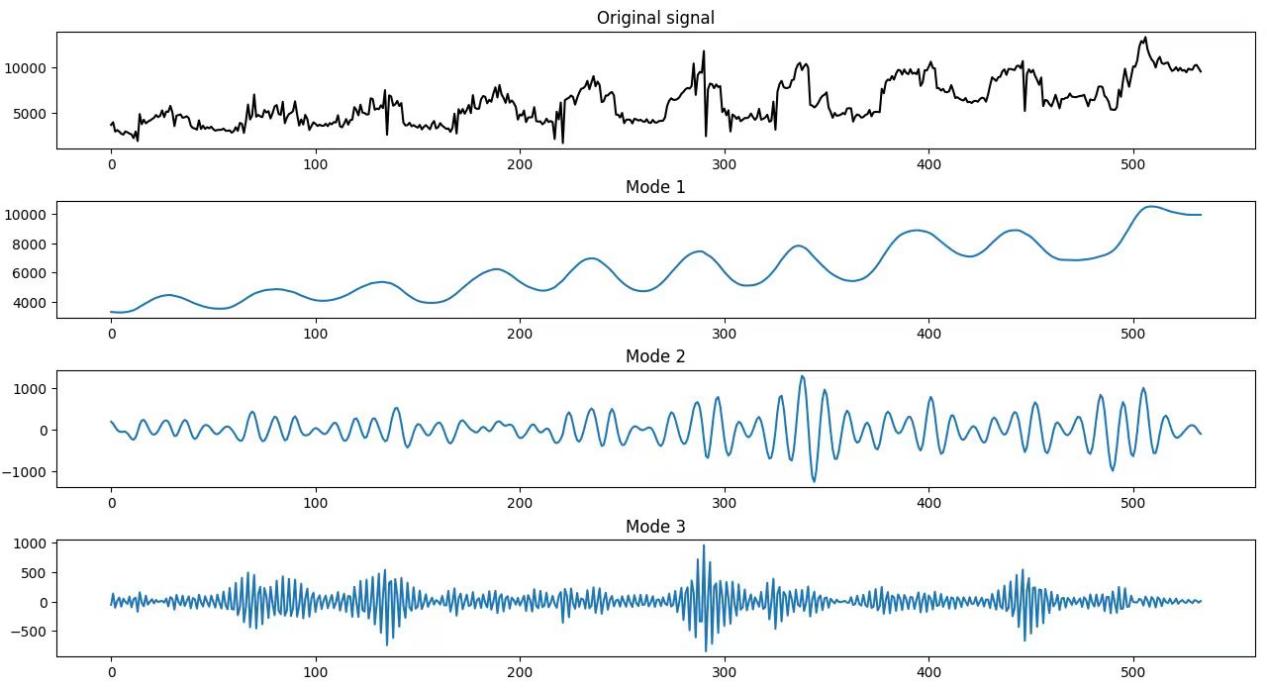
****

**（d）VMD decomposition plot for α= 2000**

****
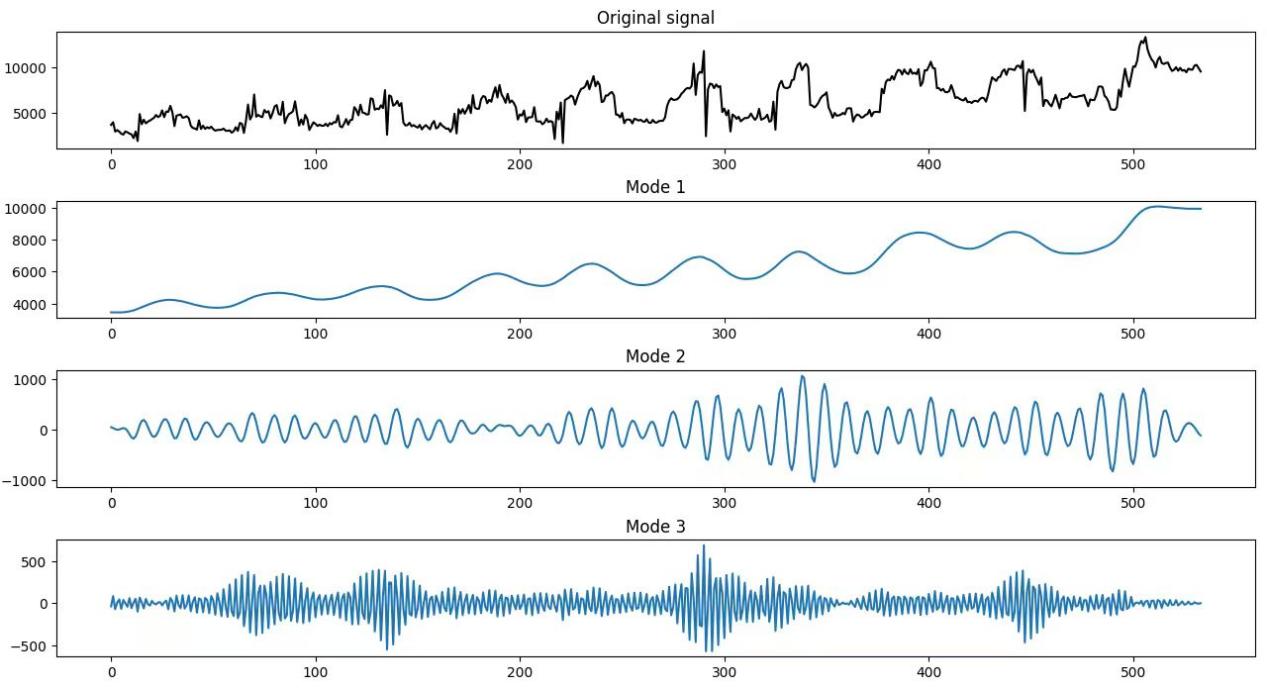
****

**（e）VMD decomposition plot for α=5000**

Figure 1. VMD decomposition plots under different **α** values.
